# Commentary: Studying a Possible Placebo Effect of an Imaginary Low-Calorie Diet

**DOI:** 10.3389/fpsyt.2020.00329

**Published:** 2020-04-29

**Authors:** Stephanie L. Dickinson, Greyson Foote, David B. Allison

**Affiliations:** ^1^Department of Epidemiology and Biostatistics, School of Public Health, Indiana University Bloomington, Bloomington, IN, United States; ^2^Department of Sociology, Indiana University Bloomington, Bloomington, IN, United States

**Keywords:** difference in nominal significance, reanalyzed data, error, correction, data analysis

While Panayotov reported very strong effects in their study of a “placebo” diet (1), the statistical analysis was performed incorrectly. To evaluate the effect of a treatment in a randomized controlled trial (RCT), the difference between groups should be tested directly rather than basing conclusions on the changes within each group separately. Conclusions in Panayotov’s manuscript are based on what has been called a DINS error or difference in nominal significance (2). A significant change from baseline within the treatment group and a non-significant change from baseline within the control group does not necessarily mean that the treatment effect was significant, i.e. that the treatment group improved significantly more than the control group. Allison and colleagues have identified DINS errors in many papers in the field of nutrition calling for their corrections (3–5).

Panayotov’s RCT tests the differential effects of participants believing they are on a low-calorie diet compared to those who know they are on an isocaloric diet (n=7 per group). Outcomes measured were body mass (kg), fat mass (%), and BMI (kg/m^2^) in the treatment and control group at the beginning of the experiment and after 8 weeks of both groups being on an isocaloric diet, where the treatment group were told they were receiving a low calorie diet. The results of the study are based on paired t-tests comparing the outcomes at baseline and 8 weeks, separately within each group. This can make treatment effects appear to be significant where they otherwise would not be.

We commend the author for sharing his data at our request, and we re-analyze the data focusing on the between-group differences to isolate the treatment effect. When we conduct t-tests on the differences (post-pre) between groups, conclusions change for one of the three outcomes (**Table 1**). Panayotov reported a p-value of < .001 for decreased fat mass in the treatment group and non-significant change (p=.21) in the control group to conclude that the treatment is better than the control. However, when we compare the decrease in the treatment group to the decrease in the control group with an independent samples t-test, we obtain a non-significant p-value of p=.105. When analyzing the % change [(post-pre)/pre], the p-value for the treatment effect on fat mass is also not significant p=.156. Treatment effects on body mass and BMI remained significant in re-analysis.

**Table 1:** Re-analysis of intervention effects.

	Trt Group	Ctrl Group	Effect size Cohen’s d	T-test	ANCOVA*
	M (SD) (n=7)	M (SD) (n=7)		p-value	p-value
Change score (Post-Pre)					
Body Mass (kg)	-9.26 (4.80)	-2.26 (4.43)	1.52	.015	.025
Fat (%)	-3.40 (0.89)	-1.43 (2.68)	1.00	.105	.096
BMI	-2.89 (1.36)	-0.99 (1.20)	1.48	.017	.023
% Change (Post-Pre)/Pre					
Body Mass (kg)	-0.08 (0.04)	-0.02 (.04)	1.63	.011	.019
%Change in Fat (%)	-0.09 (0.03)	-0.04 (.08)	0.84	.156	.142
%Change in BMI	-0.08 (0.04)	-0.03 (.04)	1.45	.020	.025

*ANCOVA includes a covariate for baseline values of each outcome.

Incidentally, in our re-analysis, we found that we were unable to replicate the results of the paired t-tests reported in Panayotov's Table 2 for the experimental group. We obtained more significant results than those reported with p=.002 and p=.001 instead of the reported p=.02 and p=.01 reported for body mass (kg) and BMI respectively.

Panayotov should re-analyze the data from his study with statistical comparisons of outcomes between groups rather than within-groups and publish corrected results.

## Author Contributions

DA: identified the error in the Panayotov paper and co-wrote the paper. SD and GF: re-analyzed the data with the correct analyses and co-wrote the paper.

## Funding

This work was funded in part by the National Institutes of Health (NIH): R25HL124208. The opinions expressed are those of the authors and not necessarily of the NIH or any other organization.

## Conflict of Interest

In the last 12 months, DA has received personal payments or promises for same from: American Society for Nutrition; American Statistical Association; Biofortis; California Walnut Commission; Columbia University; Fish & Richardson, P.C.; Frontiers Publishing; Henry Stewart Talks; IKEA; Indiana University; Laura and John Arnold Foundation; Johns Hopkins University; Law Offices of Ronald Marron; MD Anderson Cancer Center; Medical College of Wisconsin; National Institutes of Health (NIH); Sage Publishing; The Obesity Society; Tomasik, Kotin & Kasserman LLC; University of Alabama at Birmingham; University of Miami; Nestle; WW (formerly Weight Watchers International, LLC). Donations to a foundation have been made on his behalf by the Northarvest Bean Growers Association. DA is an unpaid member of the International Life Sciences Institute North America Board of Trustees. The institution of DA, SD, and GF, Indiana University, has received funds to support their research or educational activities from: NIH; Alliance for Potato Research and Education; American Federation for Aging Research; Dairy Management Inc; Herbalife; Laura and John Arnold Foundation; National Cattlemen’s Beef Association, Oxford University Press, the Sloan Foundation, The Gordan and Betty Moore Foundation, and numerous other for-profit and non-profit organizations to support the work of the School of Public Health and the university more broadly. DA’s prior institution, the University of Alabama at Birmingham, received gifts, contracts, and grants from other organizations including the Coca-Cola Company, Pepsi, and Pepper/Snapple.
